# Evaluating the Effects of BSA-Coated Gold Nanorods on Cell Migration Potential and Inflammatory Mediators in Human Dermal Fibroblasts

**DOI:** 10.3390/jfb15100284

**Published:** 2024-09-26

**Authors:** Nouf N. Mahmoud, Ayat S. Hammad, Alaya S. Al Kaabi, Hend H. Alawi, Summaiya Khatoon, Maha Al-Asmakh

**Affiliations:** 1 Faculty of Pharmacy, Al-Zaytoonah University of Jordan, Amman 11733, Jordan; 2 Department of Biomedical Sciences, College of Health Sciences, QU Health, Qatar University, Doha 2713, Qatar; 3 Biomedical Research Center, Qatar University, Doha 2713, Qatar

**Keywords:** gold nanorods, BSA, cytotoxicity, wound healing, inflammatory markers

## Abstract

Albumin-coated gold nanoparticles display potential biomedical applications, including cancer research, infection treatment, and wound healing; however, elucidating their interaction with normal cells remains an area with limited exploration. In this study, gold nanorods (GNR) were prepared and coated with bovine serum albumin (BSA) to produce GNR-BSA. The functionalized nanoparticles were characterized based on their optical absorption spectra, morphology, surface charge, and quantity of attached protein. The interaction between GNR-BSA and BSA with normal cells was investigated using human dermal fibroblasts. The cytotoxicity test indicated cell viability between ~63–95% for GNR-BSA over concentrations from 30.0 to 0.47 μg/mL and ~85–98% for BSA over concentrations from 4.0 to 0.0625 mg/mL. The impact of the GNR-BSA and BSA on cell migration potential and wound healing was assessed using scratch assay, and the modulation of cytokine release was explored by quantifying a panel of cytokines using Multiplex technology. The results indicated that GNR-BSA, at 10 μg/mL, delayed the cell migration and wound healing 24 h post-treatment compared to the BSA or the control group with an average wound closure percentage of 6% and 16% at 6 and 24 h post-treatment, respectively. Multiplex analysis revealed that while GNR-BSA reduced the release of the pro-inflammatory marker IL-12 from the activated fibroblasts 24 h post-treatment, they significantly reduced the release of IL-8 (*p* < 0.001), and CCL2 (*p* < 0.01), which are crucial for the inflammation response, cell adhesion, proliferation, migration, and angiogenesis. Although GNR-BSA exhibited relatively high cell viability towards human dermal fibroblasts and promising therapeutic applications, toxicity aspects related to cell motility and migration must be considered.

## 1. Introduction

Integrating nanotechnology with biomedical research allows the design of novel nanoplatforms for promising applications in health and biomedical sciences, such as drug/gene delivery systems, diagnostic bioimaging, therapeutic interventions, and precision medicine [1,2,3].

Gold nanoparticles (GNP) stand out for their exceptional stability and unique physicochemical properties. They can tune their electronic and optical characteristics, and their ability to be conjugated with various biomolecules/drugs/polymers allows for the modulation of biological interactions, including cellular uptake, targeting, and therapeutic effectiveness [4,5,6,7]. GNP exhibit potential applications in diagnostic imaging, drug/gene delivery, wound healing, cancer therapy, and antimicrobial and antioxidant activities. [5,8,9,10]. Non-spherical GNPs, in particular, show promising biomedical applications, such as diagnostic imaging, enhanced cellular uptake, and photothermal-based anticancer activities [11]. Gold nanorods have unique optical and electrical properties, which result in tunable optical characteristics. These properties significantly enhance their effectiveness compared to spherical GNPs in various biomedical applications [12].

Protein-based nanoplatforms, focusing on endogenous proteins like Albumin (Alb), have displayed various biomedical applications. Albumin, the dominant protein in plasma, is biocompatible and stable. It possesses different charged functional groups, hydrophobic core, and thiol groups, facilitating conjugation with many materials and molecules [13,14]. Bovine serum albumin (BSA) and human serum albumin (HAS) are frequently incorporated into hydrogels and have been shown to enhance cell adhesion and proliferation potentially. They are also commonly used as biomaterials in regenerative medicine [15,16].

Albumin is commonly conjugated with GNP to improve colloidal stability through steric stabilization and to enhance bioaccumulation by forming a protein corona that stabilizes the nanoparticles and facilitates their uptake into cells [17,18]. Due to its stability and biocompatibility, albumin has diverse applications in biomedicine, particularly in wound healing and regenerative medicine [15,16]. Introducing Alb to the GNP has significantly modulated their interaction with biological interfaces, especially in cancer, inflammatory and wound research [19,20].

Alb-coated GNP have been used to improve photothermal therapy of cancers [21] and for the delivery of chemotherapeutic agents, including paclitaxel [22], methotrexate [23], cisplatin [24], and other drugs [25]. Another study found that BSA-gold nanoclusters exhibited dose- and time-dependent cytotoxicity against three cancer cell lines: human melanoma cells, human liver cancer cells, and human cervix cancer (HeLa), and two normal cell lines that represent the vascular epithelial cells, and effectively inhibited liver cancer growth in mice [20]. Interestingly, we recently found that GNP coated with Alb had potential anticancer activity against prostate and breast cancers by inhibiting cell migration and adhesion [26]. Furthermore, Alb-coated GNP showed a potential antibiotic delivery effect and enhanced antibacterial activity against bacterial strains associated with skin infections and other infection types [27,28].

Applications of GNP in wound healing research are diverse, and their impact varies based on nanoparticles’ type, shape, size and surface functionalities [5,29]. Wound healing is a complex physiological process that involves hemostasis, inflammation, proliferation, epithelization, and re-modeling stages. Crosstalk between immune cells, fibroblasts and keratocytes with various cytokines, chemokines, and growth factors is crucial to orchestrating this interplay [30]. The modulation of cytokine release is a key mechanism underlying the influence of GNP on cell migration and wound repair. For example, hyaluronic acid-coupled GNP combined with photo modulation reduced the release of pro-inflammatory markers and increased anti-inflammatory and growth factors, thereby enhancing wound repair [31]. It is worth indicating that exploring the inhibitory effect of nanoparticles on cell motility and migration in normal cells can unveil key aspects of materials toxicity; for instance, GNP and silver nanoparticles reduced migration of human fibroblasts through modulating the deposition of extracellular matrix molecules [32]. Although Alb-conjugated GNP have been employed in various biomedical applications, comprehensive studies on their interaction with normal cells remain limited in the literature. The interaction between Alb-coated GNP and fibroblasts is influenced by multiple factors, including but not limited to the morphology and concentrations of the nanoparticles and the type of normal cells. In a previous work, BSA-coated spherical GNP were non-toxic to human fibroblasts [33], while another study revealed an accumulation of BSA-gold nanoclusters within the organs of mice, accompanied by an augmented immune response [34].

In our previous study, GNR-BSA were prepared by coating GNR with a thiolated ligand (mercapto propane sulfonate, MPS) to facilitate the self-assembly of BSA molecules onto the GNR surface [35]. These nanoparticles demonstrated significant anticancer activity against prostate and breast cancer cells, with antimetastatic effects [26].

In this study, we will further explore the biological interactions of GNR-BSA with human dermal fibroblasts by assessing cell viability, cell migration, and the modulation of cytokine release. Human fibroblasts were selected as representative cells for normal and healthy tissues due to their pivotal role in maintaining tissue integrity and contributing to the inflammation process and cancer progression [36,37].

This study will provide valuable insights into these nanoparticles’ potential toxicity and biomedical applications.

## 2. Materials and Methods

### 2.1. Synthesis of GNR-BSA

#### 2.1.1. Synthesis of GNR

Hexadecyltrimethylammonium bromide (CTAB) (Sigma-Aldrich Chemicals, St. Louis, MO, USA) and sodium oleate (NaOL) (Alpha Chemika, Mumbai, India) were used to synthesize GNR as described previously [26]. Briefly, a growth solution was prepared by treating a heated mixture of CTAB and NaOL with silver nitrate and hydrogen tetrachloroaurate (III) hydrate (HAuCl_4_) (Sigma-Aldrich Chemicals, St. Louis, MO, USA). A seeding solution was simultaneously prepared using CTAB and NaBH_4_. A small volume of seeding solution was diffused into the growth solution with L-ascorbic acid. The nanorod solution was incubated overnight, centrifuged, and collected as nanoparticle pellets.

#### 2.1.2. Surface Coating of GNR with Bovine Serum Albumin (BSA); GNR-BSA

The surface coating of nanoparticles with BSA was performed as described previously [26].

Briefly, the GNR surface underwent functionalization with Sodium 3-mercapto-1-propane sulfonate (MPS, Sigma-Aldrich Chemicals, St. Louis, MO, USA) by adding 3 mg of MPS to each 1 mL of double-centrifuged GNR (2.0 nM) in an aqueous solution for 24 h. The coated GNR obtained underwent centrifugation at 10,000 rpm for 10 min and was then suspended in ultra-pure water [26].

Bovine Serum Albumin (BSA, Sigma-Aldrich Chemicals, St. Louis, MO, USA) was introduced to the GNR-MPS at a concentration of 4 mg per 1.0 mL. pH levels were measured and adjusted to ~4 using HCl. The mixtures were magnetically stirred for 4 h at room temperature and then underwent centrifugation at 10,000 rpm for 10 min. The resulting GNP pellets were collected and stored in a refrigerator at 4 °C.

#### 2.1.3. Characterization of GNR

GNR, GNR-MPS, and GNR-BSA were analysed using a UV-Vis spectrophotometer (Shimadzu UV-1800 UV-VIS Spectrophotometer, Shimadzu, Kyoto, Japan), measuring the absorption spectrum in the range of 400–1100 nm. The nanoparticles’ zeta potentials and hydrodynamic sizes were measured using Particle Size Analyzer (The Nicomp^®^ N3000 Dynamic Light Scattering Particle Size Analyzer, Entegris, Billerica, MA, USA).

The successful surface functionalization of GNR with BSA and estimation of its concentration have been performed using Bradford Reagent (Sigma-Aldrich Chemicals, USA). GNR-BSA (~1.0 nM) was used for the quantification, and the trace amount of BSA in the supernatant was considered in the estimation. A calibration curve was produced using BSA standard solutions with varying concentrations and the absorbances were measured using a microplate reader (Synergy HTX Multi-Mode Reader, Allegiant, Las Vegas, NV, USA).

TEM imaging was performed (FEI Morgani 268, FEI, operating at 60 kV, Eindhoven, The Netherlands) to validate the size and shape of the GNR. GNR samples were prepared for TEM imaging by drying a small volume of diluted GNR over Formvar-coated copper TEM grids, with uranyl acetate stain used to enhance sample visibility.

### 2.2. In Vitro Cytotoxicity of GNR-BSA and BSA towards Normal Human Fibroblasts

#### 2.2.1. Preparation of Cell Culture

GNR-BSA cytotoxicity was assessed using human dermal fibroblasts, neonatal (HDFn) (Thermo Fisher Scientific, Waltham, MA, USA). The cells were cultured in human fibroblast expansion medium (Medium 106) and supplemented with Low Serum Growth Supplement (LSGS) (Thermo Fisher Scientific, USA). According to the manufacturer, each LSGS kit supplements a 500 mL bottle of Medium 106. Incubation occurred in a CO_2_ incubator (NuAire, Plymouth, MN, USA) at 37 °C and 5% CO_2_. Upon reaching 80–90% confluency, the cells were collected using trypsin, counted, and then cultured in a 96-well plate for the cell viability experiment.

#### 2.2.2. Cellular Viability Assay

Cellular viability was assessed after fibroblasts were exposed to GNR-BSA and BSA solution (as a positive control). Fibroblasts were cultured at a density of 10,000 cells/well in a 96-well plate and incubated for 24 h. On the day of the experiment, the medium was removed, and 100 µL of GNR-BSA (concentrations: 30, 15, 7.5, 3.75, 1.875, 0.94, 0.47 μg/mL) or BSA solution (concentrations: 4.0, 2.0, 1.0, 0.5, 0.25, 0.125, 0.0625, mg/mL), were added to the fibroblasts, followed by 24-h incubation. Cell viability was estimated by the CyQUANT™ XTT Assay kit as per the manufacturer instructions (Thermo Fisher Scientific, USA). Briefly, the XTT reagent was thawed at 37 °C and mixed with the electron coupling reagent at room temperature. To the wells of the 96-well plate, 70 μL of the prepared working solution was added, and the well was incubated for 4 h. The absorbance at 450 nm and 660 nm was recorded using a plate reader (Multiskan Sky, Thermo Fisher Scientific, USA), and the specific absorbance was calculated as follows:

(1) $$Special\;Absorbance\;=\;\left[Abs\;450nm,\;Test\;-\;Abs\;450nm,\;Blank\right]\;-\;Abs\;660nm,\;Test$$

Cell viability was calculated as a percentage of the untreated cells, and the obtained results represent averages from three independent experiments with standard deviation (±SD).

### 2.3. Fluorescence Microscopy Imaging for Cell Morphology

Fibroblasts were seeded in 3 tissue culture dishes (35 mm); the next day, one was treated with Albumin (10 µg/mL), the second with GNR-BSA (10 μg/mL), and the third was untreated as a control and incubated overnight. The medium containing the treatments was then removed, and the cells underwent two washes with PBS 1×. Cells were fixed using 4% formaldehyde applied for 15 min at room temperature, followed by two PBS washes. Then, 0.1% Triton X-100 was added for 15 min, followed by two PBS washes. Next, 1% BSA was introduced for 60 min at room temperature. After removal of the BSA, 1× of Phalloidin (Alexa Fluor 488—green color, Thermo Fisher Scientific, USA) was applied for 60 min and left overnight at 4 °C. Following Phalloidin removal, the samples were washed with PBS, and cells were stained with DAPI (HiMedia Laboratories, Kennett Square, PA, USA) for 10 min. Subsequently, the samples underwent a final wash with PBS and were observed under a fluorescent microscope (Carl Zeiss Microscope, Axiovert 40 CFL, ZEISS, Germany).

### 2.4. Cell Migration of Fibroblasts after Treatment with GNR-BSA or BSA

Human dermal fibroblasts were seeded in a 48-well plate at 70,000 cells/well density. These cells were incubated for 24 h, attaining a confluency exceeding 95%, establishing a uniform monolayer. Next, a linear scratch was created along the cell monolayer in each well using the tip of a 10.0 µL micro-pipette to activate the cells. Subsequently, excess cells and debris from the scratched monolayers were thoroughly washed with PBS. For the treatment phase, GNR-BSA and BSA in supplemented Medium 106 (350 μL) were prepared at specific concentrations (10 µg/mL of GNR-BSA and 10 µg/mL for BSA) and added to the fibroblasts.

The scratch areas were observed and photographed at 0, 4, 6, and 24 h using EVOS™ XL Core microscope (Thermo Fisher Scientific, USA).

The wound closure percentage was calculated to evaluate the cellular migration potential, representing the percentage of the initially scratched area covered by cells during a specified period and presented as mean ± SD from at least four images of each treatment and the control. The wound area was measured over time using ImageJ 1.52v^®^ (National Institutes of Health, Bethesda, MD, USA), and the migration rate was expressed by calculating the wound closure percentage at any point post-scratching [38].

(2) $$Wound\;Closure\;\left(t\right)\;\%\;=\;\frac{A(0)-A(t)}{A\left(0\right)}\;\times \;100$$

where Wound Closure (t) %= The percentage of wound closure at time (*t*).

A(0) = The wound area at time zero.

$A\left(t\right)\;=\;The\;wound\;area\;at\;\left(t\right)\;hr\;post\;scratch$.

### 2.5. Analysis of Cytokines Release Following Exposing the Fibroblast to GNR-BSA and BSA

The concentrations of a panel of cytokines released from fibroblasts were measured to evaluate further the migration, proliferation, and angiogenesis potential of activated fibroblasts upon treatment with GNR-BSA or BSA.

Fibroblasts were cultured in a 48-well plate at a density of 70,000 cells/well and incubated in a 5% CO_2_ incubator at 37 °C for 24 h, achieving a confluency exceeding 95% to form a monolayer. Subsequently, a linear scratch was carefully generated along the cell monolayer in each well to activate the cells and promote the release of cytokines, utilizing the tip of a 10 µL micro-pipette. Post-scratch, excess cells and debris from the scratched monolayers were washed twice with PBS. GNR-BSA and BSA in supplemented Medium 106 were prepared at specific concentrations (10 µg/mL of GNR-BSA and 10 µg/mL for BSA) and applied to the scratched fibroblasts. Untreated scratched and un-scratched fibroblasts served as controls. The cell culture supernatants from the samples were harvested at 4 h and 24 h, subjected to centrifugation at 4000 rpm for 10 min, and approximately 150 µL of each supernatant were carefully extracted from the midpoint of the centrifugation vial and stored at −80 °C for subsequent Multiplex analysis. The Inflammation 20-Plex Human ProcartaPlex™ Panel Multiplex kit (Thermo Fisher Scientific, USA) was utilized to quantify the concentrations of the following 20 markers: cytokines (GM-CSF, IFN alpha, IFN gamma, IL-1 alpha, IL-1 beta, IL-4, IL-6, IL-8, IL-10, IL-12p70, IL-13, IL-17A [CTLA-8], TNF alpha), chemokines (IP-10 [CXCL10], MCP-1 [CCL2], MIP-1 alpha [CCL3], MIP-1 beta [CCL4]), and cell adhesion and inflammatory response markers (ICAM-1, CD62E [E-selectin], CD62P [P-Selectin]). The Luminex xMAP technology, facilitated by the LABScan3D-Multiplex system (Catalog # LABSCNXS4, Thermo Fisher Scientific, USA), was employed to measure cytokine concentrations, allowing for the quantification of proteins from a single sample [39]. Sample preparation followed the guidelines provided by the Panel Multiplex kit manufacturer. Briefly, 1× Simplex Beads were prepared by vertexing a 50× bead vial and combining it with 1× wash buffer. Following incubation on a magnetic platter, the beads were washed, and a 96-well plate was set up. Samples were introduced, and the plate was sealed for 30 min, followed by an overnight incubation at 4 °C. The following day, the plate underwent shaking, and after repeating the washing steps, a detection antibody mixture was applied. Following a 30-min incubation, beads were rewashed, and SAPE solution was introduced. After a final round of washing, a reading buffer was added, and the plate was sealed for a 5-min incubation. Subsequently, the plate seal was removed, and the plate was analyzed on a Luminex instrument per the manufacturer’s instructions for the Multiplex analyzer. The cytokine quantification involved calculating an average of four readings from two independent experiments.

### 2.6. Ethical Approval

The materials and methods employed in this study have been reviewed and approved by the Institutional Bio-safety Committee, Qatar University. Approval No.: QU-IBC-2023/076.

### 2.7. Statistical Analysis

Statistical analysis was performed using *t*-test and one-way analysis of variance (ANOVA) by GraphPad Prism, version 9.2.0.

## 3. Results and Discussion

### 3.1. Preparation and Characterization of GNR-BSA

**Figure 1:**
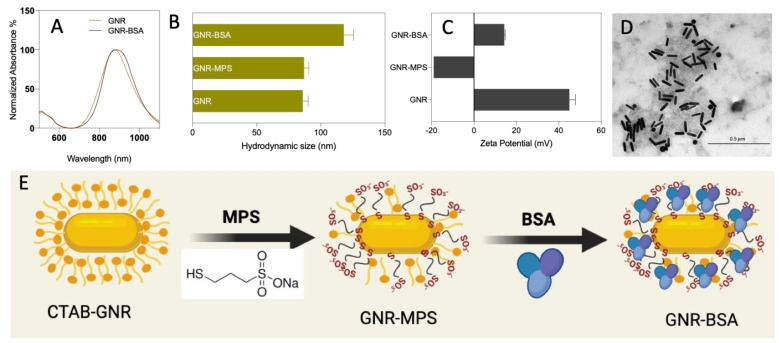
The UV-vis spectra of GNR and GNR-BSA (**A**). The hydrodynamic sizes of GNR before and after BSA coating (**B**). The zeta potential of GNR before and after BSA coating (**C**). TEM image of GNR-BSA (**D**). A Diagram illustrating the production of GNR-BSA (**E**). The results of size and zeta potentials are averaged from three independent experiments with three technical replicates.

The UV-Vis absorption spectrum of GNR revealed typical plasmon peaks at approximately 520 nm and 883 nm, respectively (Figure 1A). The average hydrodynamic sizes of GNR before and after MPS coating were 85.7 (±4.8) nm and 87.1 (±4.1) nm, respectively (Figure 1B). The surface charge of GNR was +45 (±6.3) mV, impacted by the conjugated CTAB. After coating with MPS, the charge dropped to −19.5 (±5.2) mV, indicating the successful displacement of CTAB molecules of GNR with MPS via Au-S bond (Figure 1C).

Coating GNR with the thiolated ligand (MPS) facilitates the efficient displacement of surface-bound CTAB by MPS entities through the covalent bonding (Au-S), significantly reduces nanoparticle toxicity attributed to CTAB, and enhances the colloidal stability of the nanorods [40]. Moreover, coating with MPS facilitates the self-assembly of BSA molecules onto the GNR surface [35]. MPS-GNP did not exhibit toxicity towards human keratinocytes and in an animal model [41].

GNR-BSA exhibited the longitudinal peak at 889 nm, indicating successful BSA functionalization with possible slight nanoparticle aggregation. The hydrodynamic size of GNR-BSA was 118.9 (±6.3) nm, indicating successful functionalization; however, slight nanoparticle aggregation is possible (Figure 1B). The increased zeta potential (+14.3 ± 3.1 mV) suggests effective conjugation with BSA through electrostatic attraction, with a potential contribution from hydrophobic interactions and Au-S bond (Figure 1C). TEM imaging confirmed the dimensions and morphology of GNR-BSA, revealing rod-shaped GNP with an average length and width of 89.7 (±5.6) nm and 23.2 (±5.8) nm, respectively (Figure 1D). Figure 1E illustrates coating the GNR with MPS molecules followed by conjugation with BSA molecules. The concentration of BSA bound to GNR, as measured using the Bradford assay method, was ~0.24 (±0.06) mg/mL of GNR. This corresponds to ~247 BSA molecules per gold nanorod.

Various parameters were optimized, including the concentration of GNR and BSA, temperature, pH, reaction time, and centrifugation magnitude, to achieve the most stable and efficient BSA-functionalized GNRs. BSA is composed of 583 amino acids and 17 cysteine residues [42]. The adsorption and self-assembly of BSA onto the GNR surface are justified by various mechanisms depending on the morphology and surface chemistry of GNP, the BSA concentration, and reaction pH. The hydrophobic adsorption and affinity of thiol/disulfide groups from cysteine residues to gold contribute significantly to BSA adsorption onto the surface of GNP [43]. Furthermore, electrostatic attraction between charged BSA (isoelectric point, IEP ~ 4.8) and the charged GNP also plays a significant role [35].

### 3.2. Cell Viability Assessment in Human Fibroblasts Following Treatment with GNR-BSA and BSA

**Figure 2:**
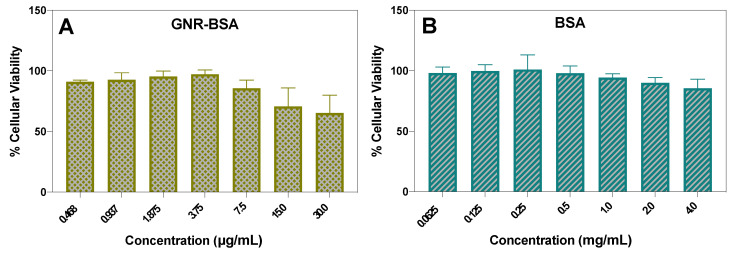
Cell viability percentages of human dermal fibroblasts 24 h post-treatment with GNR-BSA (**A**) and BSA (**B**) across various concentrations. The results are averaged from three independent experiments with three technical replicates.

The cytotoxicity assessment revealed that the cell viability of the fibroblasts averaged approximately ~63% and 70% at 30.0 and 15.0 μg/mL, respectively. In contrast, a higher cell viability of 85% was observed at other concentrations of GNR-BSA (Figure 2A). BSA demonstrates no cytotoxicity over a broad concentration range covering the highest concentrations of BSA conjugated to GNR (Figure 2B).

Our results indicate that GNR-BSA did not exhibit significant cytotoxicity across the tested concentration range compared to the positive control (BSA) and untreated cells. Notably, rod-shaped GNP were utilized in this study due to their distinguished applications in cancer, infections, and wound research compared to their spherical counterparts [12]. Furthermore, this study was conducted over various concentrations of GNR-BSA identified for therapeutic and biological responses in earlier studies. These concentrations maintained excellent nanoparticle stability when combined with cell culture media.

Although Alb-conjugated GNP has contributed to diverse applications, thorough investigations into their interaction with normal cells from various perspectives are limited in the literature.

In our previous research, GNR-BSA, prepared through direct adsorption of BSA onto the surface of CTAB-coated GNR, revealed slightly higher toxicity towards normal cells than our results, which suggests the crucial role of MPS coating in reducing the toxicity of GNR [44]. Another study indicated that spherical GNP coated with BSA were not toxic to human fibroblasts even at high concentrations [33]. Likewise, gold nanoclusters-BSA were not toxic to fibroblasts up to 10 mg/L; however, they accumulated in the liver and spleen of the experimented mice [34]. Interestingly, our recent work revealed that GNR-BSA, of the same shape and size, (have the same shape and size) showed ~17% cell viability at 30.0 μg/mL and 15.0 μg/mL against two prostate cancer cell lines (Du-145 and PC-3) and a metastatic breast cancer cell line (MDA-MB-231), and demonstrated significant antimetastatic activity. In that study, the estimated IC50 of GNR-BSA against the prostate cancer cell lines was ~9.4 g/mL, while it was ~3.0 μg/mL against the breast cancer cells [26]. These two concentrations demonstrated ≥75% cell viability towards the normal cells in our study. Peralta et al. found that GNR coated with BSA showed no cytotoxicity towards 4T1 breast cancer cells over a low range of concentrations [22]. Consequently, our findings suggest that GNR-BSA may exhibit a selective/preferential uptake into cancer cells compared to normal cells. Likewise, a recent study observed that GNP coated with BSA induced dose-dependent cytotoxicity in HeLa cells and did not reveal toxicity in normal fibroblast cells (L929 cells) [45]. The internalization of nanoparticles by tumors is often enhanced through passive targeting, utilizing the enhanced permeability and retention effect; however, coating GNP with albumin facilitates active targeting of cancer cells by binding to specific overexpressed receptors on cancer cells, such as the glycoproteins, enhancing the selective cellular internalization of nanoparticles [46]. As demonstrated in many studies, the enhanced uptake of GNP-BSA into cancer cells makes them commonly employed as drug carriers.

BSA has shown no cytotoxicity towards the cells over the concentrations used. Albumin is well-known for maintaining cell viability and growth, promoting proliferation, and supporting tissue repair. It is commonly added to cell culture media to enhance cell growth and viability. Additionally, albumin is valuable in tissue engineering and remodeling due to its bacteriostatic properties, which promote cell attachment and proliferation. Its ability to scavenge free radicals, activate neutrophils, and act as a buffer molecule further supports its role in healing processes and functional tissue remodeling. These combined properties make albumin a key component in regenerative medicine and therapeutic applications [47]. It has been demonstrated in a study that albumin promotes cell cycle progression, especially into S-phase in fibroblasts, and supports cell growth beyond its role as an amino acid source. Its beneficial properties in enhancing cell viability, proliferation, and tissue repair make it valuable in therapeutic applications [48]. Another study demonstrated the role of basic fibroblast growth factor loaded into albumin nanoparticles as a protein stabilizer, enhancing its delivery for effective wound healing and tissue regeneration [49].

Considering the potential stability of nanoparticles in cell culture media, we examined the stability of GNR-BSA across different concentrations when mixed with various media. Despite slight changes in charge and size of GNR-BSA due to the adsorption of media components, the nanoparticles maintain their stability, as evidenced by a lack of aggregation and color change. It is worth indicating that BSA is a multifunctional molecule, and even when there are changes in pH and medium components, the stability of GNP-BSA persists due to hydrophobic interactions with the gold surface and the strong Au-S affinity. Therefore, the steric repulsion the BSA-coated GNP molecules provide ensures their stability in the medium.

### 3.3. Fluorescence Microscopy Imaging for Cell Morphology

**Figure 3:**
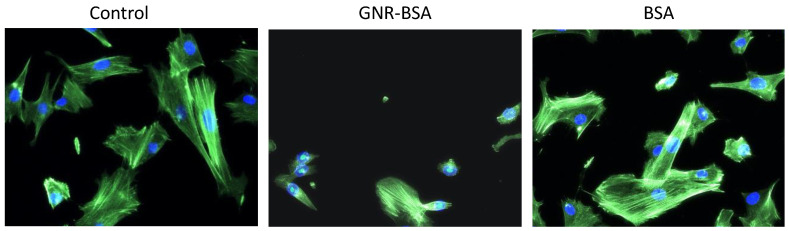
Cell morphology of human dermal fibroblasts treated with GNR-BSA, BSA, and the untreated control. The control and cells treated with BSA exhibited a normal, elongated morphology and cytoplasm. In contrast, GNR-BSA treatment led to a more rounded or oval morphology. Magnification power: 40×.

Human dermal fibroblasts typically exhibit a spindle-shaped or elongated morphology. These cells are characterized by their elongated cytoplasmic extensions and a flattened appearance [50]. This typical morphology of fibroblasts is crucial for their physiological functions, such as extracellular matrix production, tissue repair, and wound healing. In our experiments, fibroblasts treated with BSA displayed normal cell morphology comparable to untreated cells (Figure 3). However, upon exposure to GNR-BSA, the fibroblast underwent a morphological transition, adopting a more rounded or oval shape, as illustrated in Figure 3.

This alteration in cell shape is often associated with cellular responses to stress or adverse conditions and is not always associated with toxicity and could be transient. Furthermore, GNR-BSA may impact the cell membrane integrity or alter the signaling pathway, thereby influencing the cytoskeletal architecture upon cellular internalization and reorganizing the cell’s morphology. Previous research has demonstrated that cells exposed to sub-cytotoxic doses of GNP demonstrated morphological alterations accompanied by F-actin disruption in human dermal fibroblasts [51]. In another study, polymer-coated GNP induced lysosomal swelling and altered mitochondrial morphology and actin and tubulin cytoskeleton [52]. In our study, it is important to note that the concentration of GNR-BSA used in this experiment showed 75% cell viability. This suggests that while the cells retained a substantial degree of viability, the exposure to GNR-BSA at the specified concentration was sufficient to induce notable morphological changes.

### 3.4. Cell Migration Potential of Human Fibroblasts Following Treatment with GNR-BSA or BSA

The wound-healing process involves communication between various cells, mediators, and cytokines. Fibroblasts, among other cells, play a crucial role in wound healing, breaking down the fibrin clot, cell migration, and creating extracellular matrix (ECM) [30]. Cell migration is a distinctive feature in immune response, wound healing, cancer invasion, and angiogenesis. Consequently, analyzing cell migration in vitro is a valuable assay for quantifying the wound-healing potential of novel materials or interventions [38].

**Figure 4:**
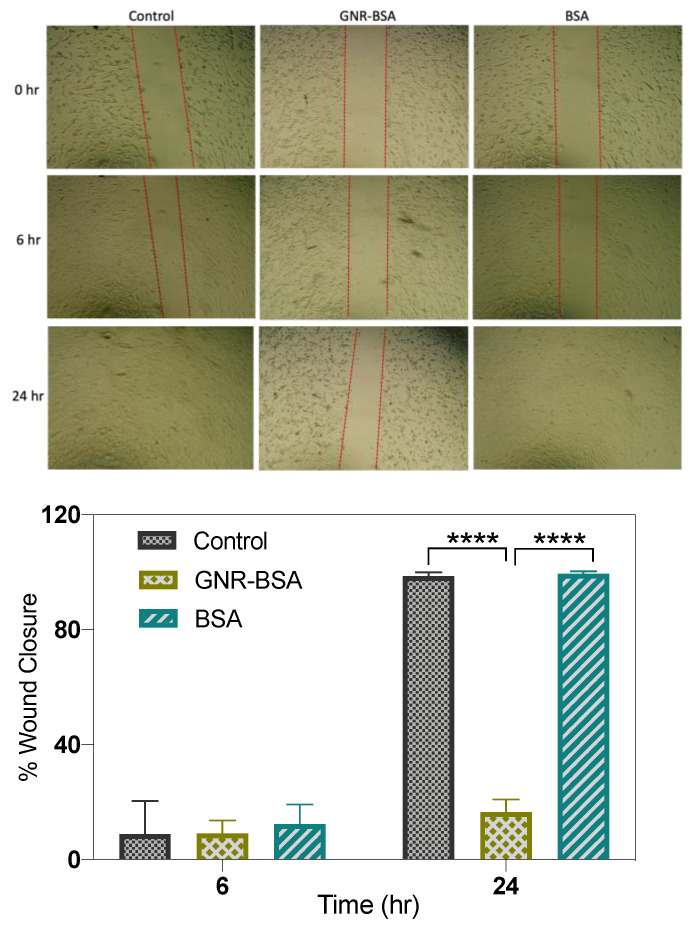
In-vitro cell migration of human dermal fibroblasts 24 and 48 h post-treatment with GNR-BSA or BSA (up). GNR-BSA treatment inhibited cell migration 24 h post-treatment compared to the BSA or control. Magnification power: 10×. Wound closure percentage of human dermal fibroblasts following treatment with GNR-BSA or BSA compared to the control (down). The results are averaged from two independent experiments with three technical replicates. **** *p* < 0.0001.

In our study, the migratory behavior of fibroblast cells was examined following treatment with GNR-BSA and its corresponding concentration of BSA using the scratch assay methodology. The concentration of GNR-BSA chosen for this study was the highest, causing ≥70% cell viability, and it was within the range of IC-50 observed in other studies for GNR-BSA against cancer cell lines. The results revealed that fibroblasts treated with GNR-BSA exhibited a significantly slower cell migration rate at 6- and 24-h post-treatment than untreated cells (Figure 4). The average percentage of wound closure was constrained, reaching 6% and 16% at 6 and 24 h, respectively, in contrast to untreated cells (Figure 4). Conversely, BSA treatment facilitated complete wound closure after 24 h compared to the control, with an average percentage of wound closure of ~12% and ~99% at 6 and 24 h post-treatment (Figure 4). Interestingly, fibroblasts displayed morphological changes 24 h post-treatment with GNR-BSA as some cells became rounded or irregularly shaped with decreased cell density compared to earlier time points.

The outcomes of the cell migration assay suggest that GNR-BSA impeded wound repair and might have induced specific alterations in the cell membrane or cytoplasm, leading to inhibited cell motility. The observed morphological changes may have negatively affected cell motility, hindering cell migration and wound closure. The effect of GNP on cell migration/wound healing depends on the nanoparticle’s properties, surface chemistries, and type of cells [5]. For example, fibroblasts exposed to varying concentrations of citrate-GNP (0.1, 1, and 10 μg/mL) exhibited reduced cell migration by modulating the ECM deposition and ECM receptors’ expression [32]. Conversely, GNP coated with quercetin have stimulated the dermal fibroblast and keratinocyte cell migration, along with upregulation of TGFβ1 protein expression [53].

Although Alb-coated GNPs have been investigated for various biomedical applications, their impact on wound healing, cell migration, and adhesion is limited in the literature. Previous studies, such as Pela et al., demonstrated that GNP conjugated with 4,6-Diamino-2-pyrimidinethiol and BSA demonstrated antibacterial activity and enhanced wound healing in cells and animal models [54]. Although albumin is often incorporated into hydrogels and dressing materials to enhance wound healing [55], integrating it into GNP has significantly modulated their therapeutic/toxicity responses. In a previous study, GNR-BSA prepared through direct adsorption of BSA onto the CTAB-coated GNR surface has impeded the dermal fibroblast cell migration [44]. Furthermore, recent findings indicate that BSA-coated GNP exhibit potential antimetastatic properties, inhibiting cellular migration and adhesion in prostate and breast cancer cells [26]. These results suggest that despite the potential of GNR-BSA as antimetastatic agents against cancer cells, careful consideration is required concerning their toxicity against normal cells.

It is worth mentioning that while the scratch assay is widely used to assess cell migration, it has inherent limitations that may affect the accuracy of the results. These include the potential influence of cell proliferation during the experiment and variations in the manual creation of the wound, leading to inconsistent wound widths. Additionally, it is challenging to distinguish between migration and proliferation contributions to wound closure without controlling for cell proliferation [56].

### 3.5. Modulation Impact of GNR-BSA and BSA on Cytokines Release from Stimulated Fibroblasts

#### 3.5.1. The Impact of Stimulation of Fibroblasts on the Modulation of Cytokine Release

**Figure 5:**
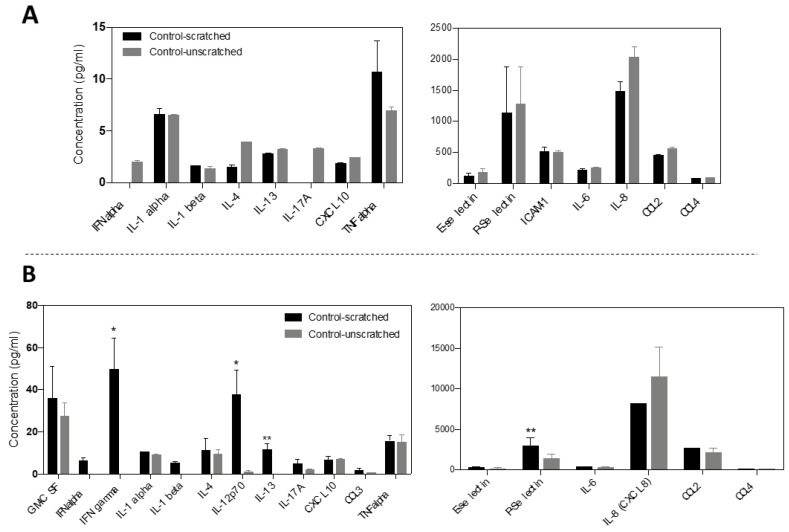
Modulation of cytokine release in stimulated fibroblasts at 4 h (**A**) and 24 h (**B**) post cell scratch. Cell scratching activated fibroblasts, leading to increased release of TNF alpha, IFN gamma and IL-12 for inflammation response. Cytokines that fall below the detection limits are excluded. The data are presented as mean ± SD. * *p* < 0.05, ** *p* < 0.01. The results are averaged from two independent experiments with three technical replicates.

Fibroblasts were stimulated through cell scratching, followed by GNR-BSA or BSA treatment. After this treatment, the early and late cytokine release was quantified at 4- and 24-h post-treatment. The efficiency of activation/stimulation of the fibroblasts was investigated by comparing the release of cytokines in the scratched cells and unscratched cells. The results indicated an early release of TNF-alpha 4 h post-fibroblasts activation (Figure 5A), followed by a significant increase in P-Selectin, IL-12, and IFN gamma markers at 24 h post-activation (*p* < 0.01, *p* < 0.05, and *p* < 0.05, respectively) (Figure 5B). Although IL-13 release was higher in the activated cells than in unscratched cells after 24 h (*p* < 0.01), although both groups exhibited similar early IL-13 release (Figure 5A,B). Notably, the release of IL-10, a potent anti-inflammatory marker, was not detected in the cells, likely due to fibroblasts not being a primary source for its release; thus, its low or absent release makes its detection challenging. Wound healing is orchestrated by various molecular mediators released from platelets, neutrophils, macrophages, and fibroblasts. Cytokines are pivotal in regulating inflammation, proliferation, angiogenesis, re-epithelization, and tissue remodeling [57,58]. Pro-inflammatory markers such as TNF alpha, IL-1 alpha, IL-1 beta, IL-6, and IL-12 initiate the early inflammatory response. As the healing process progresses into the proliferative and remodeling phases, anti-inflammatory mediators, growth factors, and chemokines help to suppress the inflammatory response and support re-epithelization, tissue repair, and angiogenesis. The transition from the inflammatory phase to the proliferation and re-epithelization phases is regulated by IL-4, IL-8, and IL-13 release [59]. Chemokines such as CCL2, CCL3, CCL4, and CCL10 regulate the inflammatory responses, proliferation and angiogenesis through the wound healing course [60], and E- and P-Selectins contribute to cell adhesion and inflammatory response [61]. Proper balance between pro-inflammatory and anti-inflammatory markers is crucial for efficient wound repair and preventing the development of chronic wounds.

Our findings revealed a rapid fibroblast activation upon stimulation, evidenced by the quick release of TNF-alpha, which initiates the inflammatory response and fibroblast chemotaxis, subsequently triggering the release of other cytokines to promote re-epithelization and ECM production [62]. Although fibroblasts are not the primary source of TNF-alpha—commonly associated with immune cells—they respond to stimuli or stress by releasing pro-inflammatory cytokines like TNF-alpha [62]. Furthermore, the findings indicate that the activated fibroblasts showed a significant increase in the release of pro-inflammatory markers, IFN gamma and IL-12, which stimulate inflammatory response and regulate the subsequent angiogenesis process and chemokine secretion [63,64]. The IL-13, essential for suppressing the initial inflammatory response, was observed 24 h post-activation [65]. The peak release of pro-inflammatory markers in vitro varies based on the marker type, the stimulus, and the experimental conditions, usually ranges from 4 to 24 h.

#### 3.5.2. The Modulation of Cytokine Release Following Treatment with GNR-BSA or BSA

**Figure 6:**
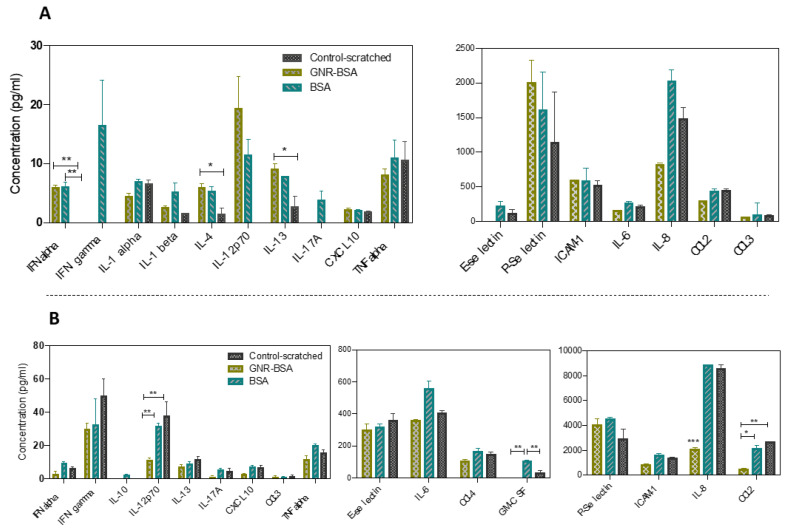
Modulation of cytokines in wounded fibroblasts treated with GNR-BSA or BSA at 4 h (**A**) and 24 h (**B**) post-treatment. GNR-BSA induced the release of IL-13 and IL-4 four hrs post-treatment, while diminished the late release of IL-8, ICAM-1, CCL2 and GM-CSF, key factors in inflammation, cell adhesion, angiogenesis, and cell migration. Cytokines that fall below the detection limits are excluded. The data are presented as mean ± SD. * *p* < 0.05, ** *p* < 0.01, *** *p* < 0.001. The results are averaged from two independent experiments with three technical replicates.

A significant reduction in CCL2 release was noted in comparison to both the BSA (*p* < 0.05) and control groups (*p* < 0.01) at 24 h post-treatment (Figure 6B). Interestingly, GNR-BSA significantly lowered the IL-8 level compared to the BSA or control 24 h post-treatment (*p* < 0.001) (Figure 6B). Furthermore, a significant decrease in GM-CSF release was observed in the GNR-BSA group compared to the BSA group (*p* < 0.01), which itself exhibited a significant increase in GM-CSF compared to the control group (*p* < 0.01) after 24 h (Figure 6B). Furthermore, a significant reduction of IL-12 release was also observed 24 h post-treatment in the GNR-BSA group compared to the BSA (*p* < 0.01) or control (*p* < 0.05) (Figure 6B).

GNR-BSA reduced the late release of the pro-inflammatory IL-12; while this modulation effect is favorable for wound healing, GNR-BSA significantly reduced the release of IL-8 chemokine 24 h post-treatment compared to other groups. IL-8 regulates the early inflammatory response and contributes to the subsequent angiogenesis, cell migration, and proliferation phases, enhancing wound healing [66]. Although excess late IL-8 production could prolong the inflammation and delay wound repair, its deficiency might impede subsequent proliferation, cell migration and re-epithelization. Several studies demonstrated that deficiency in the IL-8 activity was associated with a delay in wound re-epithelialization, angiogenesis, and, wound repair [67,68]. Moreover, GNR-BSA led to a significant reduction in CCL2 after 24 h of treatment. Stimulated fibroblasts produce chemokines that regulate epithelialization, tissue remodeling, and angiogenesis. Among chemokines, CCL2 chemokine contributes to the inflammation response and promotes angiogenesis [60]. Although the early release of CCL2 in wound healing is essential for inflammatory response, its delayed release might impact proliferation, re-epithelization, and regulation of ECM production and may prolong the inflammatory phase. Yuko et al. revealed that the topical application of CCL2 chemokine was effective for wound repair by enhancing angiogenesis and collagen production [69]. Hence, diminished secretion of IL-8 and CCL2 markers after 24 h of GNR-BSA treatment may have contributed to the observed deceleration in cell migration of wounded fibroblasts in our study. Interestingly, BSA, but not GNR-BSA, enhanced the release of GM-CSF; this cytokine contributes to cell proliferation and re-epithelization, which supports the favorable impact of BSA on wound healing [70]. Furthermore, a slight but insignificant release of IL-10 was detected in the BSA group, suggesting its favorable wound-healing effect.

The cytokine production modulation by GNP depends on the nanoparticle’s type and properties. For instance, IL-6, IL-12, and TNF alpha cytokines decreased after applying citrate-GNP to human dermal fibroblasts and epidermal keratinocytes [71]. Although various studies have explored the effect of GNP on cytokine release, research on the impact of albumin-coated GNP, in particular, on cytokine release, is limited.

#### 3.5.3. The Impact of Time on the Cytokine Release among Different Treatments

**Figure 7:**
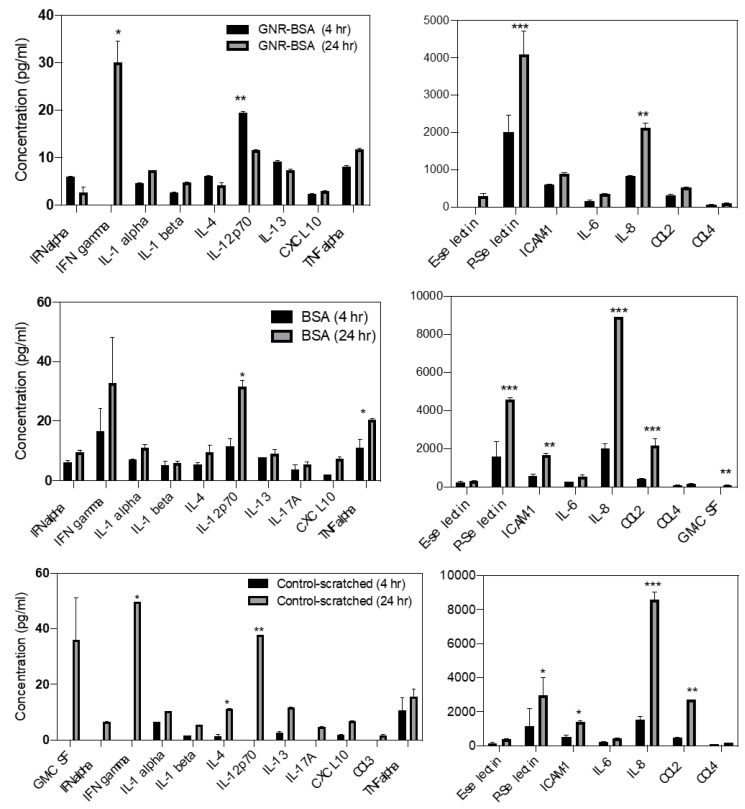
The impact of time on cytokine modulation (4 h vs. 24 h) in fibroblasts treated with GNR-BSA, BSA, and untreated cells. The release of ICAM-1, CCL4, and GM-CSF cytokines did not increase at 24 h compared to 4 h of GNR-BSA treatment when compared to BSA or the control. * *p* < 0.05, ** *p* < 0.01, *** *p* < 0.001. The results are averaged from two independent experiments with three technical replicates.

The impact of time on cytokine release was examined within each group. The results indicate a significant elevation in the release of IL-8 was found at 24 h compared to 4 h across all groups (*p* < 0.001). IL-8 is implicated in proliferation, cell migration, re-epithelization, and inflammation response (Figure 7). However, its release in the GNR-BSA group after 24 h was significantly lower than in the BSA or control groups (Figure 6B). Likewise, the release of CCL2 and ICAM-1, involved in cell adhesion, angiogenesis, and migration, increased after 24 h compared to 4 h in the BSA (*p* < 0.001) and control (*p* < 0.01) groups, but not in the GNR-BSA group (Figure 7). Notably, there was an increase in the release of IL-12, known for enhancing the inflammatory response, and IFN gamma, which exhibits an anti-angiogenic effect, after 24 h compared to 4 h in both the BSA (*p* < 0.05) and control (*p* < 0.01) groups (Figure 7). Still, this was not observed in the GNR-BSA group, as the concentration of IL-12 was elevated at 4 h compared to other groups (Figure 6A). Although IL-12 is crucial for inflammatory response and early-phase proliferation, its persistent increase might delay wound healing by delaying cell migration and angiogenesis. A significant increase in GM-CSF was observed in the BSA (*p* < 0.01) and control (*p* < 0.05) groups at 24 h compared to 4 h; however, GM-CSF was not detected in the GNR-BSA group over 24 h post-treatment (Figure 7).

These findings support the earlier results in Section 3.5.2, indicating significant modulation in the late release of IL-8 and CCL2, which contribute to cell adhesion, migration, epithelization, and angiogenesis in the GNR-BSA group compared to other groups, this could potentially impact the wound process. The contribution of GM-CSF is possible since it was not detected in the GNR-BSA group compared to the BSA or control group. Nevertheless, a significant reduction in the late release of IL-12 was observed in the GNR-BSA group, which might prevent the late persistent inflammatory response.

## 4. Conclusions

Integrating nanotechnology with medicine and biomedical science necessitates a thorough understanding of the nano-bio interface to explore potential therapeutic merits and toxicity aspects of nanoplatforms for advancing this field. In this study, gold nanorods decorated with BSA did not induce significant cytotoxicity against human dermal fibroblasts; however, they delayed cell migration and impeded wound healing. This impact on wound healing was associated with a significant modulation in the release of IL-8 chemokine, CCL2, and IL-12 markers, crucial for the inflammatory response and cell adhesion, proliferation, migration, and angiogenesis. Although GNR-BSA exhibited high cell viability towards human dermal fibroblasts, they revealed possible toxicity related to cell motility and migration toxicity. The overall results indicate that while these nanoparticles could be effective as a potential antimetastatic agent in cancer therapy, their toxicity against normal cells and possibilities of targeting techniques toward cancer cells should be considered.

Such comprehensive research would provide deeper insights into the complex interactions at the nano-bio interface and contribute to developing safer and more effective nanomedicine strategies.

## Data Availability

The original contributions presented in the study are included in the article, further inquiries can be directed to the corresponding authors.
